# Synthesis and Characterization of Multi-Walled Carbon Nanotube/Graphene Nanoplatelet Hybrid Film for Flexible Strain Sensors

**DOI:** 10.3390/nano8100786

**Published:** 2018-10-04

**Authors:** JianRen Huang, Shiuh-Chuan Her, XiaoXiang Yang, MaNan Zhi

**Affiliations:** 1 Department of Mechanical Engineering, Yuan Ze University, Chung-Li 320, Taiwan; chinafzhjr@gmail.com (J.R.H.); zhimnlucky@163.com (M.N.Z.); 2 School of Mechanical Engineering and Automation, Fuzhou University, Fuzhou 350108, China; yangxx@fzu.edu.cn; 3 Vice President Office, Quanzhou Normal University, Quanzhou 362000, China

**Keywords:** graphene nanoplatelet, multi-walled carbon nanotube, hybrid film, vacuum filtration, strain sensing

## Abstract

Graphene nanoplatelet (GNP) and multi-walled carbon nanotube (MWCNT) hybrid films were prepared with the aid of surfactant Triton X-100 and sonication through a vacuum filtration process. The influence of GNP content ranging from 0 to 50 wt.% on the mechanical and electrical properties was investigated using the tensile test and Hall effect measurement, respectively. It showed that the tensile strength of the hybrid film is decreasing with the increase of the GNP content while the electrical conductivity exhibits an opposite trend. The effectiveness of the MWCNT/GNP hybrid film as a strain sensor is presented. The specimen is subjected to a flexural loading, and the electrical resistance measured by a two-point probe method is found to be function of applied strain. Experimental results demonstrate that there are two different linear strain-sensing stages (0–0.2% and 0.2–1%) in the resistance of the hybrid film with applied strain. The strain sensitivity is increasing with the increase of the GNP content. In addition, the repeatability and stability of the strain sensitivity of the hybrid film were conformed through the cyclic loading–unloading tests. The MWCNT/GNP hybrid film shows promising application for strain sensing.

## 1. Introduction

Since the discoveries of carbon nanotubes (CNT) by Iijima [1] and graphene nanoplatelets (GNP) by Novoselov et al. [2], they have received a great attention as raw materials for the development of nanomaterials due to their excellent thermal, electrical and mechanical properties, low density and high specific surface area [3]. Nowadays, enormous efforts have been devoted to the use of CNT and graphene in various applications, such as energy storage [4,5], field effect transistors (FETs) [6], electrodes [7,8] and sensors [9,10]. Thin films or paper-like materials consisting of GNP or CNT have drawn extensive attention and they are being widely employed for supercapacitors [11], pressure sensors [12], monitoring cure behavior of polymer composite [13], flexible temperature sensors [14], and as reinforcing fillers in polymers [15,16,17]. These free-standing thin films are cohesively bound by van der Waals interactions among entangled CNTs and GNPs. The main idea behind the fabrication of thin film is to utilize the excellent properties of individual GNP and CNT in macroscopic form. This thin film is advantageous to facilitate easier handling of GNPs and CNTs and to improve the capability of using GNPs and CNTs in industry [18]. These thin films are suitable for both lightweight structural and functional applications.

Flexible strain sensors have been highly desirable in applications such as electronic skin, structural health monitoring, and robot sensors in recent years. CNT and GNP are applicable for piezoresistive strain sensors and have been of great interest among researchers. Lu et al. [19] employed a flexible GNP/epoxy strain sensor to monitor the deformation and damage in structural composites. Liu et al. [20] reported a highly reliable strain sensor based on graphene composite film with layered structure. Moriche et al. [21] studied the strain monitoring mechanism of GNPs incorporated into epoxy matrix. Sanli et al. [22] investigated the piezoresistive performance of strain-sensitive MWCNT/epoxy nanocomposites. Wang et al. [23] utilized a CNT composite film as a strain sensor to monitor biaxial strain under tensile tests. Wang et al. [24] developed a new processing technique of MWCNT strain sensors with tunable strain gauge factors. Natarajan et al. [25] examined the efficiency and effectiveness in terms of piezoresistive properties of natural rubber composites based on MWCNT, carbon black and their mixtures (hybrid). The change of relative resistance, was found to be as much as ∼1300 at around 120% elongation. Boland et al. [26] incorporated graphene into a lightly cross-linked polysilicone, resulting in a change of its electromechanical properties substantially. These nanocomposites were sensitive electromechanical sensors with gauge factors >500 that can measure pulse, blood pressure, and even the impact associated with the footsteps of a small spider. Li. et al. [27] fabricated flexible and electrical conductive carbon cotton/polydimethylsiloxane composites by vacuum-assisted infusion for highly sensitive pressure sensor. The flexible pressure sensor exhibited a maximum sensitivity of 6.04 $KPa^{-1}$ in a wide working pressure up to 700 kPa. Samad et al. [28] developed a graphene foam/polydimethylsiloxane flexible sensor to sense both compressive and bending strains in the form of change in electrical resistance. They found that resistances can be increased to 120% and 52% of its original value by applying a 30% compressive strain and bending a sample to a radius of 1 mm, respectively. Samad et al. [29] fabricated freestanding, mechanically stable, and highly electrically conductive graphene foam with two-step facile, adaptable and scalable techniques. They demonstrated the capability of graphene foam as strain/pressure sensor for both high and low strains and pressures with tunable densities.

The potential applications of CNTs and GNPs are limited because CNTs are easy to entangle and agglomerate due to the large aspect ratio and GNPs also tend to restack due to van der Waals and strong interactions. One of the most efficient ways to avoid the agglomeration is to incorporate CNTs with GNPs to produce a nanocomposite material or a hybrid. CNTs can bridge adjacent graphene layers and retard graphene interlayer stacking, resulting in an increased contact surface area between GNPs. Hybrid CNT/graphene films are typically bonded by π–π interaction, which can induce functionalization due to the difference in geometry between the GNP and the CNT. Apart from the non-covalent interaction, covalent bonds and hydrogen bonds have also been used to construct hybrid graphene and CNT nanomaterials, which are confirmed to be of ultrahigh strength, modulus, electrical conductivity and thermal performance [17]. Most of the existing literature, as mentioned above, studied the sensing capability of the carbon nanomaterials such as MWCNT and GNP individually. Relatively few studies have been reported on electrical and mechanical properties of hybrid films. This work seeks to explore the piezoresistive behavior and strain-sensing ability of the MWCNT/GNP hybrid film. The synergistic effect of MWCNT and GNP on the electrical conductivity is presented. These are the novelty and originality of present work. In this study, MWCNT/GNP hybrid films were fabricated by vacuum filtration of mixed dispersion with varied MWCNT-to-GNP weight ratios. A series of hybrid films with different amounts of MWCNTs and GNPs were prepared. The loading of GNPs varied from 0 wt.% to 50 wt.% and that for MWCNTs was 100 wt.% to 50 wt.%, respectively. The effect of GNP content on mechanical properties, electrical conductivity and strain-sensing performance of hybrid films are investigated. A controllable strain sensitivity of the hybrid film can be achieved by varying the GNP content. It is important in understanding the MWCNT/GNP hybrid films so as to further improve their properties for end applications.

## 2. Experiments

In this study, two types of nanomaterials, a multi-walled carbon nanotube (MWCNT) and a graphene nanoplatelet (GNP), were used to fabricate the hybrid films with different weight ratios.

### 2.1. Materials

GNPs were purchased from UChees Co. (Taiwan, China) with 1~10 nm thickness, 0.5–20 μm lateral dimension and surface area of 400–700 m^2^/g. MWCNTs, grown by CVD, were purchased from Conjutek Co. Taiwan with the diameter in the range of 10–50 nm, length of 100–200 μm, surface area of 400–700 $m^{2}$/g, and purity >98.5%. Both of GNPs and MWCNTs were used as received without any modification.

### 2.2. Film Preparation

**Figure 1:**
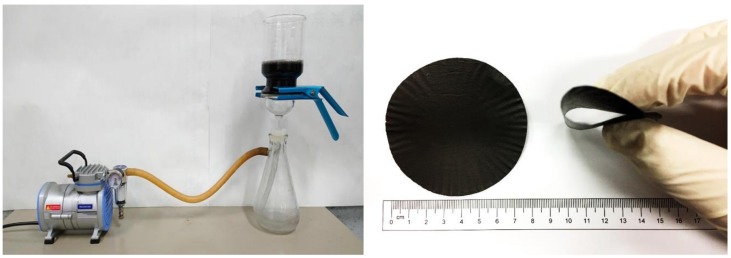
Experimental setup of the vacuum filtration and as-prepared hybrid film.

MWCNTs and GNPs have strong tendencies to form bundles and aggregate together because of their high surface area and the strong van der Waals interaction. They are also hydrophobic and have poor solubility in aqueous solutions [30]. In this study, the MWCNT/GNP hybrid films were prepared with the aid of surfactant Triton X-100. The molecular structure of surfactant Triton X-100 (Big Sun Chemical Corp., New Taipei City, Taiwan) contains a hydrophilic polyethylene oxide group and hydrocarbon lipophilic (or hydrophobic group), which improves the dispersibility of MWCNTs and GNPs in aqueous solution [31]. The hybrid films with different weight ratios of MWCNT and GNP were prepared using the following process. The total mass of MWCNTs and GNPs was held constant at 0.16 g. Surfactant Triton X-100 with the weight of 5 g were dissolved in 500 ml deionized water and dispersed by a sonicate tip (Q700, Qsonica L.L.C., Newtown, CT, USA) for 30 min at 30 W. The sonicator was operated at pulse mode (10 s on and 20 s off). Then, a total mass 0.16 g of MWCNTs and GNPs with a desired weight ratio were added to the suspension and dispersed by a sonicate tip for 3h at 30 W. Upon completion of the dispersion process, the MWCNT/GNP suspension was filtered through a Polytetrafluoroethylene (PTFE) microporous membrane (pore size 0.45 μm, diameter 90 mm) by a vacuum filtration. The experimental setup of the vacuum filtration is shown in Figure 1. After filtration, the hybrid film was peeled off from the filter membrane and washed by a large amount of isopropyl alcohol to remove any residual surfactant. The film was dried in a vacuum oven preheated to 40 °C for 12h. The typical thickness of the hybrid film was 60–80 μm. Following the same process, a series of hybrid films with GNP weight percentage ranging from 0 wt.% to 50 wt.% were fabricated to investigate the effect of GNP on the mechanical and electrical properties. In this study, hybrid films with GNP weight percentages of 0%, 10%, 20%, 30%, 40% and 50% were denoted as GNP-0, GNP-10, GNP-20, GNP-30, GNP-40 and GNP-50, respectively.

### 2.3. Morphology

Field emission scanning electron microscope (JSM-7600F, Jeol Ltd., Tokyo, Japan) was performed to characterize the surface morphology and cross-section view of the hybrid film. An accelerating voltage of 10 kV and a working distance of 5–10 mm were adopted to generate the field emission scanning electron microscope (FESEM) images of the hybrid film. The samples were sputter-coated with a conductive gold layer before taken the image.

**Figure 2:**
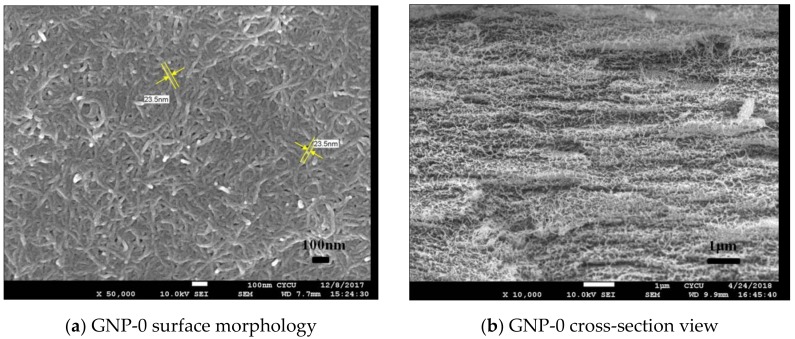

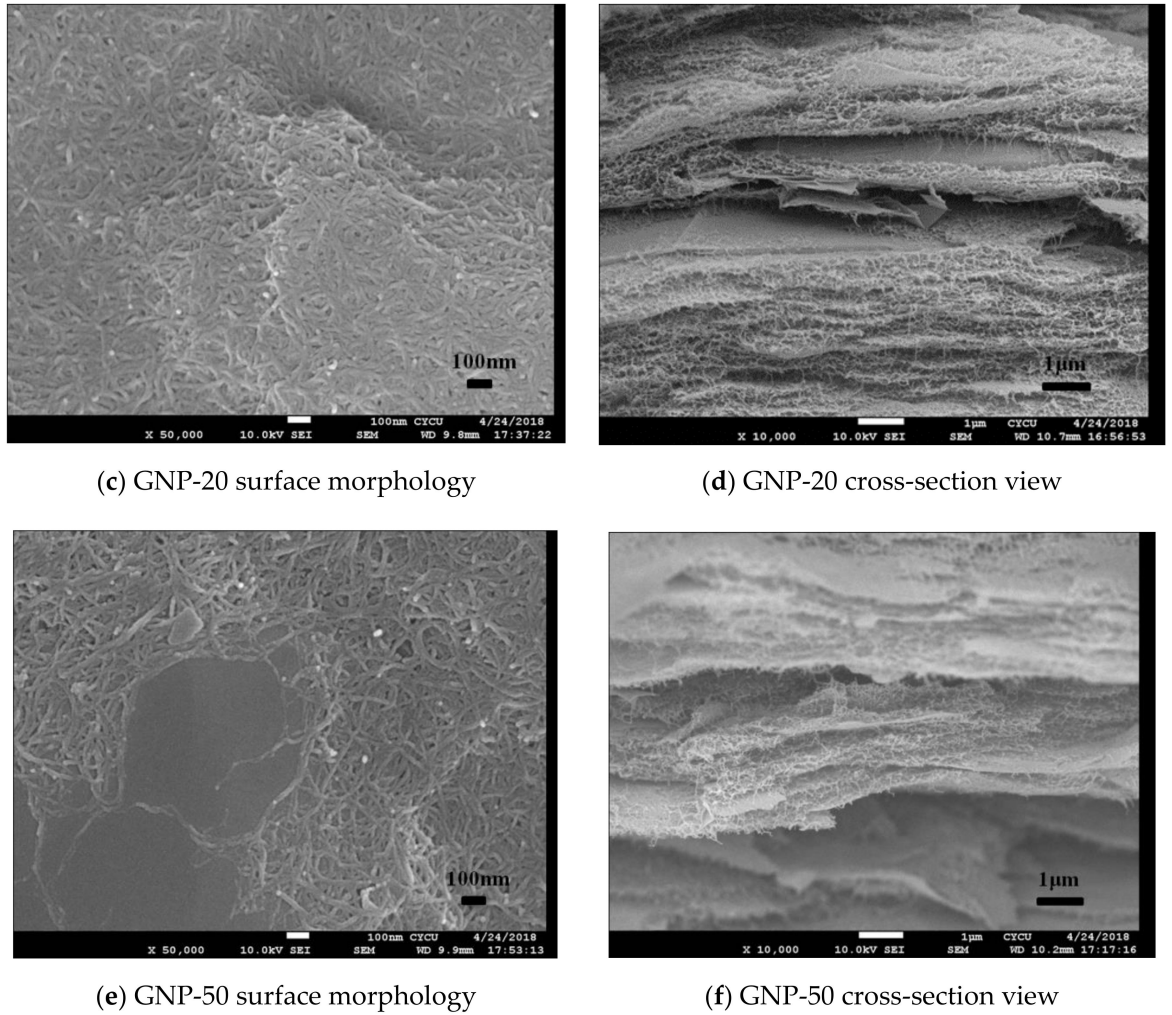
SEM images of hybrid films (**a**) surface morphology of GNP-0; (**b**) cross-section view of GNP-0; (**c**) surface morphology of GNP-20; (**d**) cross-section view of GNP-20; (**e**) surface morphology of GNP-50; (**f**) cross-section view of GNP-50.

A typical MWCNT/GNP hybrid film is shown in Figure 1 It can be seen that the film is highly flexible, which can be rolled up or bear small radius bending without any damage or fracture. The surface morphology and cross-section view of the hybrid film with 0 wt.% (GNP-0), 20 wt.% (GNP-20) and 50 wt.% (GNP-50) of GNP are presented in Figure 2. The surface morphology of the film GNP-0 (0 wt.% GNP and 100 wt.% MWCNT) exhibits homogenous and densely packed mass of randomly oriented MWCNTs without any agglomeration, and this orientation gives rise to its isotropic properties as shown in Figure 2a. For the hybrid film GNP-20 (20 wt.% GNP and 80 wt.% MWCNT), most of the GNPs are covered by the MWCNTs as shown in Figure 2c. As the content of GNP increases, some of the GNPs can be observed on the top of MWCNTs as shown in Figure 2e for hybrid film GNP-50 (50 wt.% GNP and 50 wt.% MWCNT). GNPs and MWCNTs are uniformly dispersed and highly entangled with each other. From the cross-section SEM images shown in Figure 2b,d and f, MWCNTs and GNPs are successfully deposited to form densely packed film with layered structure. Similar layered structure with MWCNTs distributed between GNP sheets in the flexible GNP/MWCNT film using as a high performance supercapacitor was also reported by Lu et al. [11]. It can be attributed to the filtration-induced directional flow during the fabrication process. Under the vacuum filtration pressure, the 2D GNP tended to self-adjust their basal planes parallel to the filter membrane plane due to the large aspect ratio of GNP sheets, resulting in significant alignment of GNP sheets [32]. Clearly, GNP sheets served as the supporters to hold the MWCNTs in-between, generating a more compact and aligned structure of hybrid films. Graphene sheets uniformly spread on MWCNTs and alternately stacked layer structure are observed. With the increase of GNP content, the long and tortuous MWCNTs are embedded between the GNP layers, which can prevent the aggregation of GNPs. It appears that MWCNTs were preferentially oriented and bridged the gap between the GNP layers.

## 3. Results and Discussions

### 3.1. Mechanical Properties

The mechanical properties of hybrid film were evaluated by uniaxial tensile testing. The specimens were cut into a rectangle strip with 30 mm in length and 10 mm in width. Tests were conducted using a universal testing machine with 200 N load cell at a constant cross-head speed of 0.5 mm/min. To reveal the reproducibility of the results, three samples were fabricated and tested for each hybrid film. The experimental results reported in this work are the averaged values.

**Figure 3:**
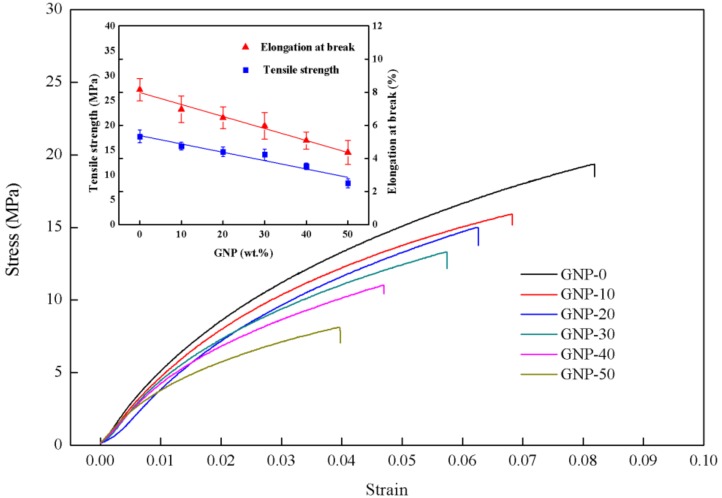
Stress-strain curves of the hybrid films with different weight percentage of graphene nanoplatelets (GNP).

Figure 3 plots the typical stress–strain curves of the hybrid films with different GNP contents ranging from 0 wt.% to 50 wt.%. The mechanical properties including the Young’s modulus, tensile strength and fracture strain can be extracted from the stress–strain curve. Table 1 lists the tensile strength and fracture strain of the hybrid films. Based on published literature, the mechanical properties of CNT buckypaper, tensile strength of 2–94 MPa, Young’s modulus of 2.1 MPa to 3.84 GPa, and fracture strain of 0.3–2% have been reported [18]. Present results are within the range of typical MWCNT buckypaper. It can be observed that both the tensile strength and fracture strain are decreasing with the increase of the GNP content as shown in the inset of Figure 3. Tensile strength and fracture strain of GNP-0 (0 wt.% GNP and 100 wt.% MWCNT) are 17 MPa and 8.2%, respectively, which are 105% and 86% higher than that of GNP-50 (50 wt.% GNP and 50 wt.% MWCNT). These results can be inferred from the SEM images that the MWCNT bundles exhibit not only strong Van der Waals and π–π interactions but also mechanical interlocking through entanglements and form a strong robust network [33]. However, the graphene sheets are mainly assembled by an in-plane contacting via Van der Waals forces without being strongly inter-connected. Thus, the tensile strength of the hybrid film is decreasing as the MWCNT content decreases.

**Figure 4:**
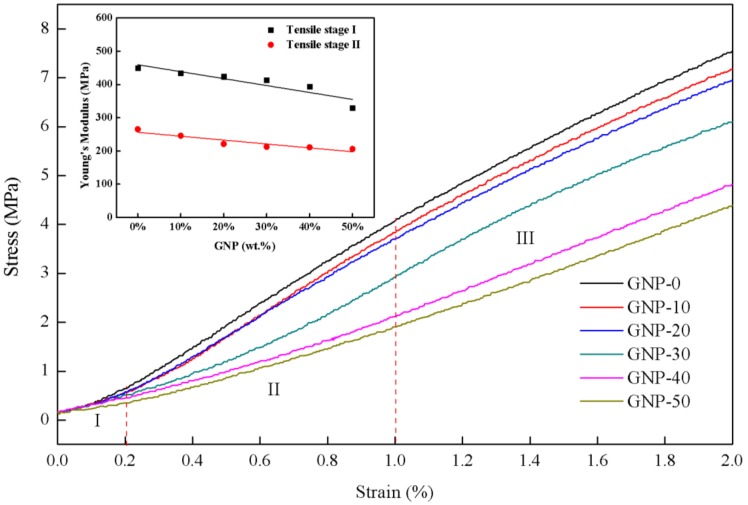
Enlarged stress –strain curve of MWCNT/GNP hybrid films.

The enlarged stress–strain curves as shown in Figure 4 can be divided into three stages. In stage I (strain ranging from 0 to 0.2%), the wavy MWCNTs are first straightened upon tensile loading, causing little change in stress with linear stress-strain relationship [17]. In stage II (strain ranging from 0.2 to 1.0%), the deformation happens under fairly law stress and the joints between MWCNTs and GNPs inside the film are stretched resulting in a higher elastic modulus which is analogized to the disentanglement of polymer chain [18]. In stage III (strain >1.0%), with further stretching of the hybrid film, interlock between GNPs and MWCNTs gradually fails and the network becomes loosely, a non-linear stress-strain relationship is observed. At the initial stage of loading, significant straightening took place both in the GNPs [34] and MWCNTs leading to alignment along the tensile direction, after which the curves became almost linear at higher strains. Elastic moduli of the hybrid films in stages I and II of the tensile testing are shown in the inset of Figure 4. The elastic modulus of the hybrid film in stage II is higher than that of stage I by approximately 200 MPa. It appears that GNPs were easier to be straightened due to the slippage of the overlapped GNPs; i.e., it is more flexible than MWCNTs which were interlocked with each other. The tensile strength and Young’s modulus of the hybrid film measured as a function of GNP content are shown in Figure 3 and Figure 4, respectively. Both properties consistently increased with decreasing GNP content, indicating the dominance of MWCNT on mechanical properties of the hybrid film.

### 3.2. Electrical Properties

The sheet resistivity of the hybrid film was measured by the Hall effect (Ecopia HMS-3000). To demonstrate the reproducibility of the experimental results, three specimens with square shape (10 × 10 mm) were cut from different locations of the hybrid film and tested. The average value was reported with standard deviation.

**Figure 5:**
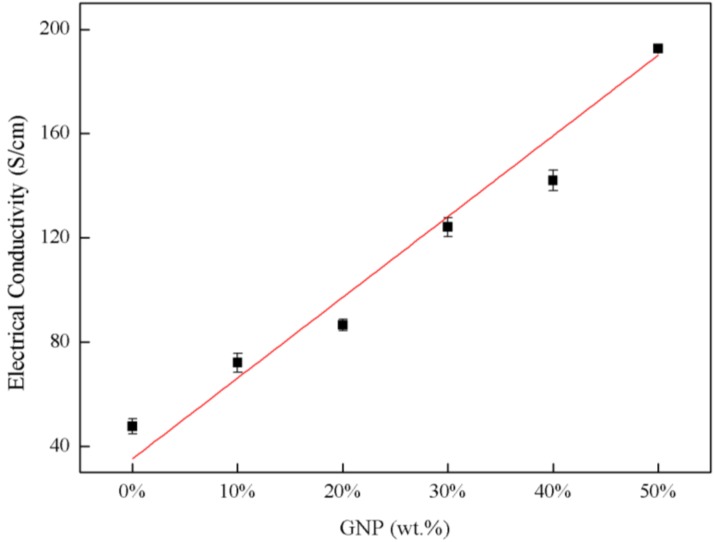
Electrical conductivity of MWCNT/GNP hybrid films with different weight percentages of GNP.

Table 2 lists the electrical resistivity and conductivity of the hybrid film with different weight percentages of GNP. It can be seen that the conductivity of the hybrid film is increasing with the increase of the content of GNP as shown in Figure 5. While both MWCNTs and GNPs are highly conductive, GNPs are more conductive for two reasons. Firstly, their two-dimensional nature results in a better connectivity and so a greater choice of conductive paths for electrons to flow through. Secondly, their planar nature allows them to pack more closely than MWCNTs, giving lower porosity [35]. Thus, GNP is the dominant factor on the electrical property of the hybrid film. A remarkable increase in the electrical conductivity from 47.72 S/cm to 192.60 S/cm was observed when the GNP content was increased from 0 to 50 wt.%. The conductivity of the hybrid film GNP-50 was enhanced by 304% in comparison with the GNP-0, due to the formation of 3D conductive networks [36]. The hybrid film exhibits a well-stacked layered structure throughout the cross section. The MWCNT network bridges the gap between the GNPs. Larger lateral dimension of GNP acts as a strong holder while MWCNT serves as a wire to connect GNP. The conductivity of the hybrid film depends on the conductive network formed by the MWCNTs and the inherent conductivity of GNPs. At a low weight fraction of GNP, MWCNT and GNP are not close-packed to form effective conductive pathways in the hybrid film. The overlap of MWCNTs introduces larger interfacial resistance that further decreases the conductivity of the hybrid film with too much MWCNTs in the grapheme layer. When the fractions of GNPs were increased, the percolated network of MWCNTs and GNPs was formed which provided efficiently conductive pathways for electron transfer in the hybrid film. The decrease in the sheet resistivity by incorporation of GNPs demonstrates that two-dimensional GNPs provide a more efficient percolating network than one-dimensional MWCNTs. Furthermore, GNP worked as strong holders with a large surface area to support contact between the MWCNT and GNP, resulting in a further reduction of the contact resistance. In the MWCNT-dominated hybrid film, a pronounced synergistic effect on conductivity can be observed. The electrical conductivity is related to both in-plane and through-thickness conduction of electrons. It is clear to see from Figure 2 that 1-D MWCNTs act as bridges to connect 2-D GNPs and provide additional channels for the electron transfer within the hybrid film. This leads to a decreased electrical resistance and may be considered as the major reason for the synergistic effect of the MWCNT and GNP hybrid films. In addition, high electrical conductivity of GNP in the basal plane enhances the synergistic effect on electrical conductivity.

### 3.3. Self-Strain Sensing Properties

**Figure 6:**
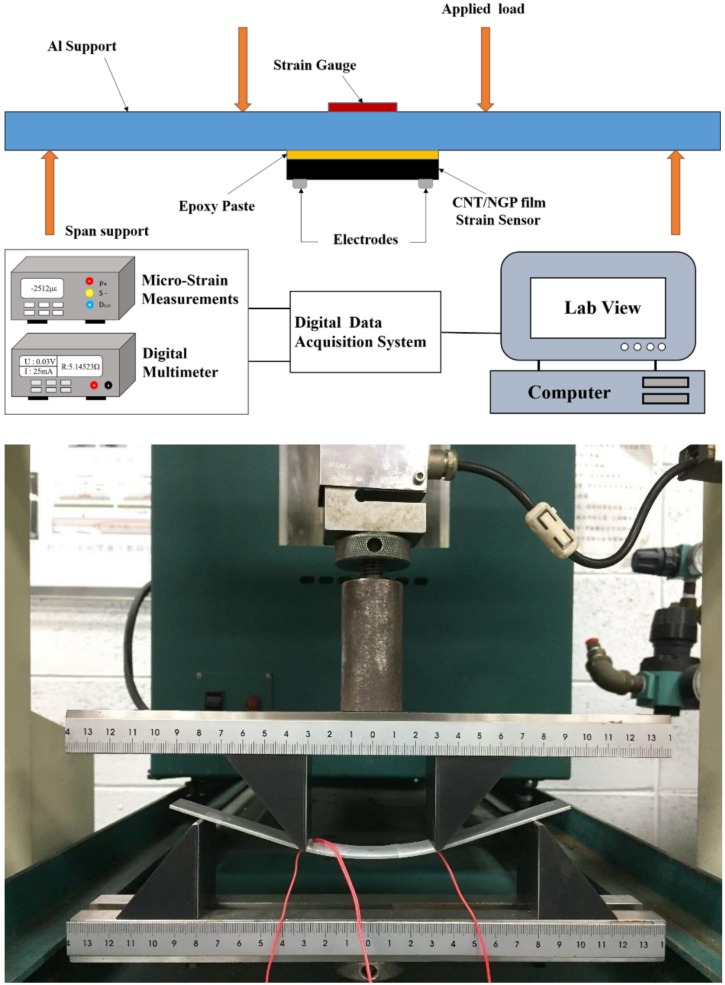
Schematic diagram and experimental setup of the four-point-bending test.

The prepared hybrid film was cut into a rectangular strip (30 × 10 mm) for evaluation of the piezoresistive response and sensing performance. The strain-monitoring capability of the hybrid film sensor was tested in a flexural test. Electrical resistance and mechanical strain during the test were measured simultaneously by a digital Multimeter (Keithley 2450) and strain gauge, respectively. The hybrid film sensor was attached to the center of an Al (Al6064-T6) test specimen (dimensions: 200 × 19 × 2 mm) using epoxy to make perfect bonding between the specimen and hybrid film sensor. When the load was applied on the Al specimen, the hybrid film bonded through high strength epoxy and the metallic strain gauge experienced the same strain. Two copper electrodes were adhered to the hybrid film sensor at a distance of 25 mm using silver paste to minimize the contact resistance. The electrical resistance in monitoring tests was measured by the two-point method due to the simplicity of the method regarding scalability to real applications [21]. Resistivity and strain data were recorded by the digital data acquisition system (cDAQ-9174 NI) through the Lab VIEW software. In this work, a four-point-bending test was conducted to study the piezoresistive behavior of the hybrid film sensor. The spans between the two inner points and two outer points are 60 mm and 120 mm, respectively. A schematic diagram and experimental setup of the four-point-bending test are shown in Figure 6.

The addition of GNPs increases the conductivity of the hybrid film as described in Section 3.2. Four-point-bending tests were performed to monitor the electrical resistance change of the hybrid film with different GNP contents induced by the strain. Gauge factor is an important parameter which can be used to describe the sensitivity of the strain sensor. It is defined as the ratio of the normalized electrical resistance and strain induced in the sensor as follows.

(1) $$\;\mathrm{GF}=\frac{\Delta R/R_{0}}{\varepsilon }\;$$

where ΔR is the resistance change with strain, $R_{0}$ is the initial resistance prior to straining, ε is the applied strain.

**Figure 7:**
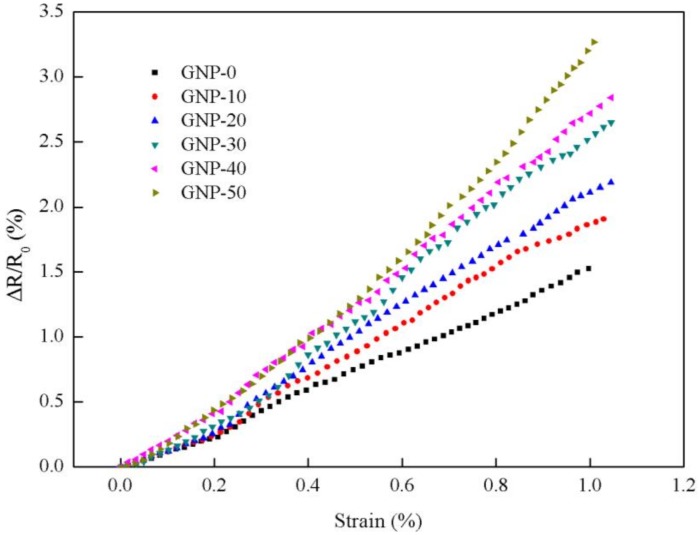
Normalized resistance change increases with the increase of the strain.

**Figure 8:**
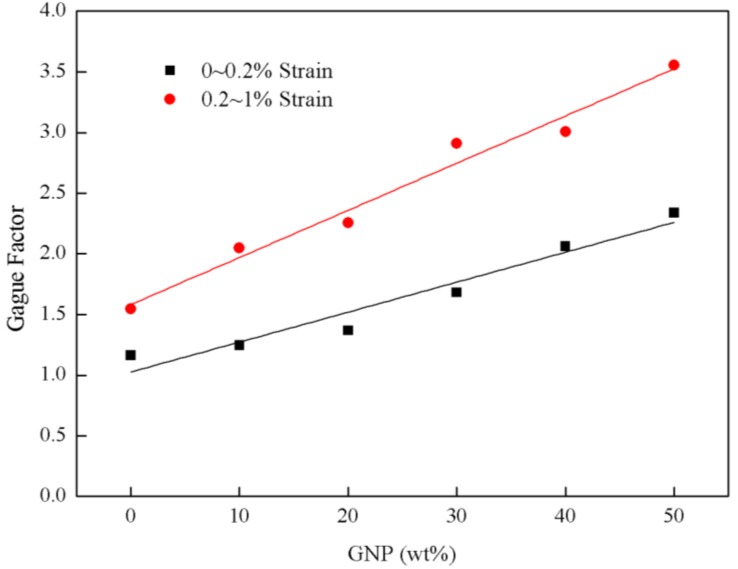
Gauge factor for different strain stages.

**Figure 9:**
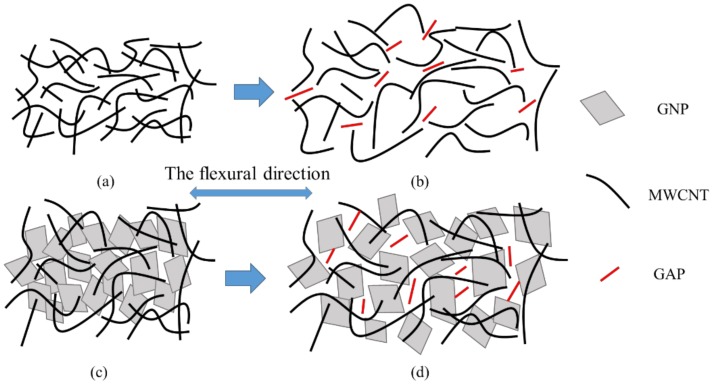
Schematic representation of microstructure changes in hybrid films subjected to mechanical strain. (**a**) MWCNT film; (**b**) Stretching of MWCNT film under flexural strain; (**c**) MWCNT/GNP hybrid film; (**d**) Stretching of MWCNT/GNP hybrid film under flexural strain.

Representative normalized resistance–strain curves of the experimental results are plotted in Figure 7 for various GNP contents ranging from 0 to 50 wt.%. It can be observed that the normalized resistance behaves in positive piezoresistive trend, i.e., the normalized resistance change monotonic increases with the increase of the strain. Moreover, the normalized resistance of the hybrid film is increasing with the increase of GNP content. For the increase of the resistance curve, an evident change occurs around at the strain of 0.2%. The whole curve can be divided into two stages. In stage 1 (strain range 0–0.2%), the increase of the resistance tends to be linear with a small slope. In stage II (strain range 0.2–1%), the resistance change exhibits a linear relationship with a large slope. The slope of the curve represents the gauge factor of the hybrid film which can be used to characterize the strain sensitivity of the hybrid film sensor. The gauge factors of the hybrid films with different GNP contents for stage I and II are listed in Table 3. It can be observed that the gauge factor is increasing with the increase of the GNP content as shown in Figure 8. Furthermore, gauge factor in stage I is larger than that of stage II. As the GNP content increases from 0 wt.% to 50 wt.%, the gauge factor increases from 1.16 to 2.34 in stage I, and increases from 1.54 to 3.56 in stage II. The mechanism corresponding to the increase of the resistance in the two stages can be explained as follows. The resistance of the hybrid film can be attributed to three main aspects, namely, contact resistance, tunneling resistance and intrinsic resistance. In stage I, the gauge factor is mainly affected by intrinsic resistance, the relative displacements of MWCNT and GNP are small, the wavy carbon nanotubes are straightened under strain due to its large flexibility, and a smaller gauge factor is acquired. However, in stage II, the normalized resistance change (ΔR/R_0_) of the hybrid film is mainly relied on the contact and tunneling resistances of adjacent nanomaterial sheets. The conductivity between neighboring flakes is determined by their overlap area and the contact resistance [37]. The assumption in the sensitivity change of MWCNT/GNP hybrid films can be further explained by the schematic diagram shown in Figure 9. Once a mechanical strain is applied to the hybrid film, the overlap area between neighboring flakes becomes smaller and the gap distance becomes larger, which results in an increase of the tunneling pathway between adjacent nanoplatelets so the tunneling resistance increases. In the process of mechanical loading, the tunneling resistance instead of the contact resistance becomes the dominant factor of the resistance. In addition, the more the GNP content, the more easily the conductive path gets disrupted by external strains, which results in higher strain sensitivity. Similar results were reported by Lu et al. [38]. They found that the sensitivity of the GNP/epoxy sensor was varied along with the applied strain and can be separated to three strain regions (0–0.2%), (0.2–0.6%) and (0.6–1.2%), respectively. The gauge factors of the GNP/epoxy sensor with 1.58 vol.% of GNP corresponding to these three strain regions were 2.53, 3.77 and 4.69, respectively.

**Figure 10:**
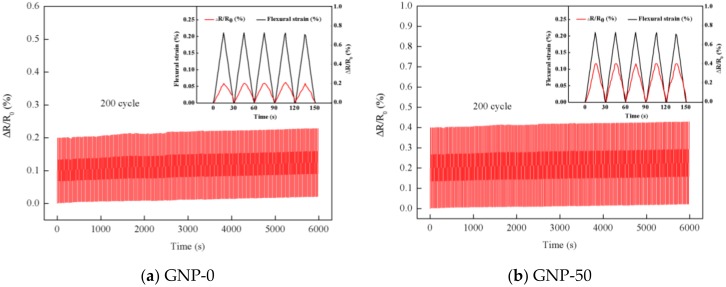
Normalized resistance change and mechanical strain of the hybrid film under cyclic loading-unloading test (**a**) 0 wt.% GNP-0 (**b**) 50 wt.% GNP-50.

To investigate the stability, reversibility and reliability of the hybrid film sensor, the specimens were subjected to 200 cyclic loading–unloading tests. This test aimed to monitor the electric resistance response of the hybrid film under cyclic loading. The dynamic responses of the normalized resistance change and mechanical strain of hybrid films with 0 wt.% (GNP-0) and 50 wt.% (GNP-50) of GNP are plotted in Figure 10a,b, respectively. It can be observed that there is no obvious change during the 200 cycling tests for the hybrid film sensors. This demonstrates that the high durability and stability of the hybrid film sensor. Some researchers [39,40] also did the cycle loading–unloading tests for stability of the GNP/epoxy sensors, they are stable under certain cycles, but the cycles they tested were as low as 50 or even several cycles.

## 4. Conclusions

MWCNT/GNP hybrid films were prepared with the aid of surfactant Triton X-100 and sonication through vacuum filtration process. SEM images show that MWCNTs and GNPs are successfully deposited to form densely packed film with layered structure. The effect of GNP content ranging from 0 to 50 wt.% on the mechanical and electrical properties of the hybrid films were characterized using the tensile test and Hall effect measurements, respectively. It can be observed that both the tensile strength and fracture strain are decreasing with the increase of GNP content. The electrical conductivity is increasing from 47.7 S/cm to 192.6 S/cm as the GNP loading increases from 0 to 50 wt.%. A series experimental tests were conducted to study the piezoresistive behavior and the strain-sensing capability of the hybrid film. The gauge factor defined as the ratio of relative change in resistance to applied strain was used to characterize the sensitivity of the strain sensor. There are two different linear strain-sensing stages (0–0.2% and 0.2%–1%) in the resistance of the hybrid film with applied strain. The gauge factor increases from 1.164 to 2.236 as the GNP loading increases from 0 to 50 wt.% in the strain-sensing range 0–0.2%. Moreover, the repeatability and stability of the strain sensitivity of the hybrid film were conformed through the cyclic loading and unloading tests. From the results obtained, it is demonstrated that the MWCNT/GNP hybrid film is very suitable for strain sensing.

## Figures and Tables

**Table 1:** Mechanical properties of the hybrid film with different weight percentage of graphene nanoplatelets (GNP).

Hybrid Film	Tensile Strength (MPa)	Fracture Strain (%)
GNP-0	17 ± 1.3	8.2 ± 0.7
GNP-10	16 ± 0. 8	7.0 ± 0.8
GNP-20	15 ± 1.0	6.5 ± 0.7
GNP-30	14 ± 1.1	6.0 ± 0.8
GNP-40	12 ± 0.6	5.1 ± 0.5
GNP-50	8.3 ± 0.9	4.4 ± 0.7

**Table 2:** Electrical properties of hybrid film with different weight percentage of GNP.

GNP wt.%	Resistivity (Ω · cm)	Conductivity (S/cm)
GNP-0	2.1×10^−2^ ± 1.4×10^−3^	48 ± 3.0
GNP-10	1.4×10^−2^ ± 7.0×10^−4^	72 ± 3.6
GNP-20	1.2×10^−2^± 2.9×10^−4^	87 ± 2.1
GNP-30	8.1×10^−2^ ± 2.4×10^−4^	124 ± 3.7
GNP-40	7.0×10^−3^ ± 2.0×10^−4^	142 ± 4.0
GNP-50	5.2×10^−3^ ± 3.0×10^−5^	193 ± 1.1

**Table 3:** Gauge factor for different strain stage.

Gauge factor						
GNP wt%	0%	10%	20%	30%	40%	50%
0~0.2% strain	1.2	1.3	1.4	1.7	2.1	2.3
0.2~1% strain	1.5	2.1	2.3	2.9	3.0	3.6
